# Preparation and Biological Properties of Oligonucleotide-Functionalized Virus-like Particles

**DOI:** 10.1021/acs.biomac.3c00178

**Published:** 2023-05-31

**Authors:** Robert Hincapie, Sonia Bhattacharya, Parisa Keshavarz-Joud, Asheley P. Chapman, Stephen N. Crooke, M. G. Finn

**Affiliations:** ^†^School of Chemistry and Biochemistry, ^‡^School of Biological Sciences, Georgia Institute of Technology, 901 Atlantic Drive, Atlanta, Georgia 30332, United States

## Abstract

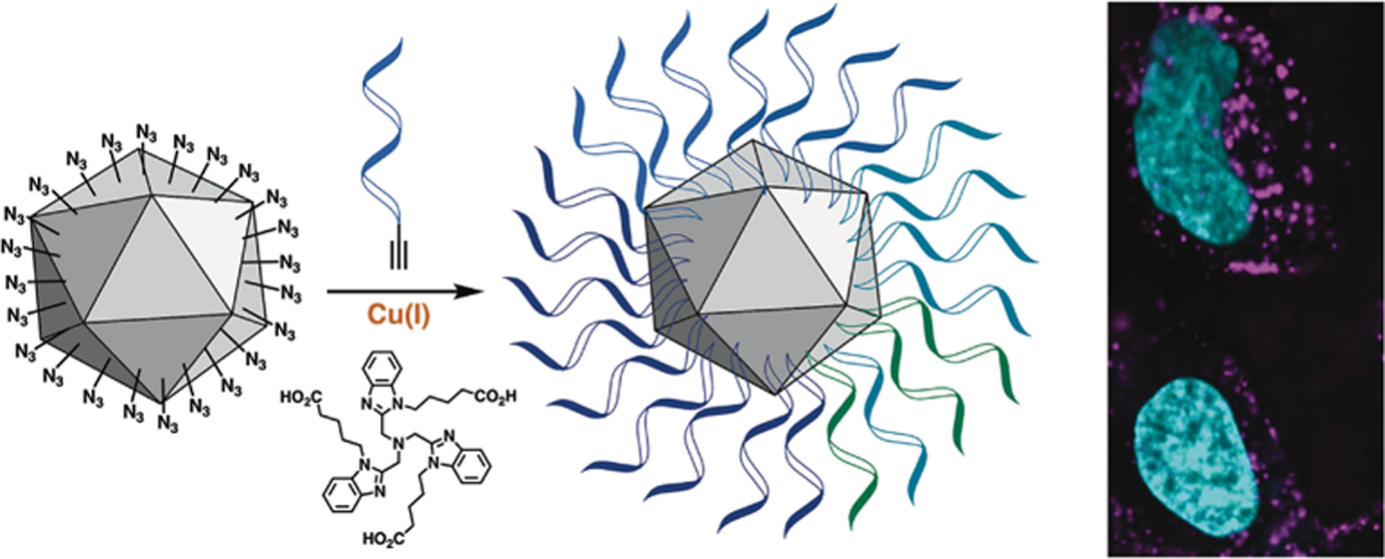

Oligonucleotides are powerful molecules for programming function and assembly. When arrayed on nanoparticle scaffolds in high density, the resulting molecules, spherical nucleic acids (SNAs), become imbued with unique properties. We used the copper-catalyzed azide–alkyne cycloaddition to graft oligonucleotides on Qβ virus-like particles to see if such structures also gain SNA-like behavior. Copper-binding ligands were shown to promote the click reaction without degrading oligonucleotide substrates. Reactions were first optimized with a small-molecule fluorogenic reporter and were then applied to the more challenging synthesis of polyvalent protein nanoparticle–oligonucleotide conjugates. The resulting particles exhibited the enhanced cellular uptake and protection from nuclease-mediated oligonucleotide cleavage characteristic of SNAs, had similar residence time in the liver relative to unmodified particles, and were somewhat shielded from immune recognition, resulting in nearly 10-fold lower antibody titers relative to unmodified particles. Oligonucleotide-functionalized virus-like particles thus provide an interesting option for protein nanoparticle-mediated delivery of functional molecules.

## Introduction

Oligonucleotides are programmable molecules with an extraordinary range of applications. Dense clusters of oligonucleotides on nanoparticles (dubbed “spherical nucleic acids,” or SNAs, and most commonly based on inorganic or liposomal cores) have dramatically different properties than their separate components. Some functions are expected from their polyvalent nature, such as binding with much greater affinity to complementary sequences and being much more resistant to degradation by nucleases, thereby enabling sensitive detection or gene regulation. Other functional capabilities are less easy to predict, such as readily entering a variety of cells by engaging scavenger cell-surface receptors,^1−3^ leading to uses in diagnostics and target detection,^4^ chemotherapy,^5^ immunology,^6,7^ and immunotherapy.^8,9^

Virus-like particles (VLPs) and other protein nanoparticles have biological properties (such as immunogenicity) and capabilities (such as the packaging of other functional biomolecules) that are not shared by the traditional SNA platforms. It is therefore of interest to see if dense oligonucleotide functionalization has as dramatic effects on these structures as well. Indeed, recent efforts by Mirkin and colleagues have demonstrated that protein core SNAs (ProSNAs) exhibit many of the useful characteristics of inorganic nanoparticle-based analogues while retaining properties such as biocompatibility and functional enzyme activity.^10−12^

In particular, the innate ability of VLPs to activate TLR-7 signaling by packaging bacterial RNA, as well as their immunologically favorable biodistribution and trafficking properties make oligonucleotide-decorated particles interesting candidates for biological application. Earlier work on this theme includes a number of reports focused on engineering sequence-dependent assembly or organization;^13−16^ and, more recently, examples of DNA origami used to template tobacco mosaic virus assembly^17,18^ and aptamer-directed virus capsids for in vitro imaging^19,20^ and drug delivery.^21^ The earliest efforts used amine or thiol-reactive chemistry to install DNA–protein linkages;^13^ copper(I)-catalyzed azide–alkyne cycloaddition (CuAAC) has also been employed to prepare oligonucleotide conjugates of MS2 (27–38 oligonucleotides per 28 nm diameter particle), using an air-sensitive copper(I) source.^22^ In this case, the authors noted that increasing the concentration of oligonucleotide past a certain threshold led to fewer oligonucleotides attached per particle, and attributed this observation to reaction inhibition by the negatively charged oligonucleotide. More recently, oligonucleotides have been site-selectively conjugated to engineered adeno-associated virus (AAV) by strain-promoted alkyne–azide cycloaddition (SpAAC); the resulting conjugates had limited density (up to 60 per 25 nm diameter capsid, actual value not determined) and relied on an electrostatically bound lipid agent to impart resistance against serum neutralization.^23^ A relevant precedent describes the surface decoration of nanoparticles from the E2 protein with immunostimulatory CpG oligonucleotides (16 ± 5 CpG per 30 nm diameter particle) via thiol alkylation. These particles showed enhanced trafficking to lymph nodes and enhanced uptake by dendritic cells and antigen-presenting cells within the lymph node compared to control particles lacking the oligodeoxynucleotide.^24^

In some of these earlier reports, protein nanoparticle–oligonucleotide conjugates were prepared with a limited loading of oligonucleotide (one or fewer oligonucleotides per viral capsid subunit), falling short of the display density required to impart conjugates with SNA-like properties (>1.5 pmol DNA/cm^2^),^25^ and substantially below the DNA densities typically achieved using AuNPs (ca. 20–60, and recently up to 100 pmol DNA/cm^2^).^2,26−28^ We explored the further use of the CuAAC reaction as a convenient alternative, the fast rate and strong driving force of which should be useful for demanding ligations such as formation of high-density covalent surface arrays on large protein nanoparticles. We thereby prepared VLPs displaying a shell of oligonucleotides (designated V-SNAs) with 100–300 copies of 18- or 30-mer oligodeoxynucleotides per particle. Our V-SNAs achieve a similar loading density to that of other ProSNAs in the literature,^12,26,29^ and approximately half of a typical density for similarly sized AuNPs (Table 1). Relative to unlabeled particles, these oligo-coated VLPs exhibited the enhanced cellular uptake characteristic of SNAs, decreased antigenicity, and similar residence time in the liver before clearance.

**Table 1:** Reports of Oligonucleotide-Protein Nanoparticle Conjugates, Compared to Typical Au–SNAs and Liposomal SNA Preparations^e^

entry	platform	function or description	size (nm)^a^	#strands	oligo length^b^	DNA density (pmol/cm^2^)	refs
1	Qβ	GGT repeat	14	100–270	18	6.7–18.2	this work
2	Qβ	GGT repeat	14	100–140	30	6.7–9.2	this work
3	AAV	Cell binding	12.5	≤60^c^	35	≤5.1	(23)
4	E2	CpG	15	16–21	20	0.9–1.2	(24, 30)
5	MS2	aptamer	14	20–60	41	1.3–4.0	(15, 19)
6	MS2	assembly	14	20	20	1.3	(21)
7	MS2	aptamer	14	54	37	3.6	(20)
8	MS2	CpG	14	38	20	1.8	(22)
9	HBc	CpG	14	≤120^d^	20	≤8.1	(31)
10	Qβ	GGT repeat	14	20	20	1.3	(13)
11	Qβ	assembly	14	190	18	12.8	(14)
12	catalase	assembly	14 × 8.5 × 7.5	44	18	16.9	(10, 32)
13	β-gal	GGT repeat	9 × 7.5 × 9	30	34	3.7	(11, 26)
14	LacOx	assembly	6	12	35	4.4	(29)
15	AuNP	n.d.^e^	15	600	25	35.2	(27)
16	lipoNP	T30	16	70	30	4.1	(33)

a Radius of approximately spherical nanoparticles, or dimensions otherwise.

b Number of nucleotides.

c Assuming 100% of possible sites loaded with oligonucleotide.

d Assuming 50% of possible sites loaded with oligonucleotide (clickable handle incorporated at dimer interface via genome engineering, so the highest loading is one oligonucleotide per two subunits).

e n.d. = not described.

## Materials and Methods

### Fluorogenic CuAAC

Coumarin azide,^57^ BimC_4_E,^35^ BimC_4_A,^35^ and THPTA^58^ were prepared as previously described. Copper(II) sulfate and copper-binding ligands were mixed in ratios as described in the main text (Table 2). To an aliquot of copper:ligand complex were added coumarin azide (100 μM final concentration), oligonucleotide-alkyne (20 μM final concentration), and 10× potassium phosphate buffer (1× final concentration). Reactions were initiated by the addition of sodium ascorbate (10 mM final concentration), followed by immediate inversion and incubation at 40 °C for 1 h. Reactions were monitored by fluorescence of the coumarin triazole product (λ_ex_ 404 nm, λ_em_ 477 nm) using a Varioskan Flash (Thermo Fisher Scientific) plate reader. The degree of reactivity was assessed by endpoint measurement (taking an aliquot of the reaction mixture from a capped reaction vessel) or by continuous monitoring in a plate reader held at 40 °C. Note: premixed complexes of BimC_4_E or BimC_4_A and CuSO_4_ were partially insoluble in water, requiring up to 20% DMSO and dilution (final concentration of BimC_4_E < ∼200 μM, final concentration of BimC_4_A < ∼2.5 mM) to maintain solubility. In the absence of copper, BimC_4_A was quite water-soluble (at least 50 mM), but BimC_4_E required dilution to ca. 0.1–0.25 mM.

**Table 2:**
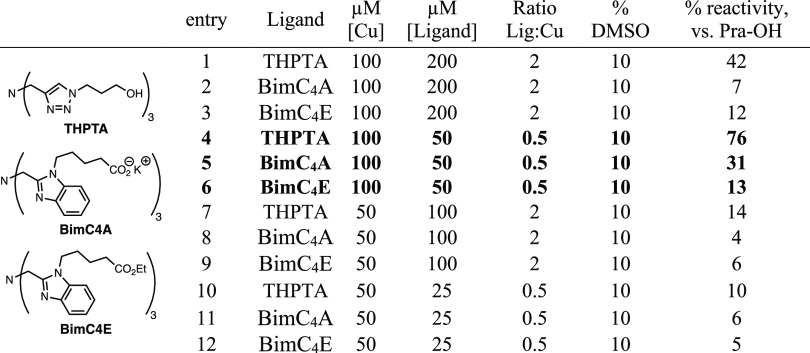
CuAAC between Fluorogenic Azide and Alkyne-Labeled Oligonucleotides, Promoted by Ligand THPTA, BimC_4_A, or BimC_4_E^a^

a Extent of reactivity was determined by fluorescence maxima following CuAAC (λ_ex_ = 404 nm, λ_em_ = 477 nm). Conditions: 100 μM coumarin azide, 20 μM alkyne, 10 mM sodium ascorbate, 40 °C, 1 h.

### Analysis of DNA Damage by Densitometry

The extent of oligonucleotide protection for “off-particle” reactions during CuAAC was determined by denaturing agarose gel electrophoresis directly on the reaction mixtures without purification. Lane intensities were analyzed using ImageJ and normalized relative to untreated DNA to determine the extent of DNA protection for each ligand during CuAAC (Figure S1).

### Expression and Purification of VLPs

BL21 (DE3) chemically competent *Escherichia coli* cells (Lucigen) were transformed with the pCDF-CP(WT-Qβ) plasmid according to the manufacturer’s protocol. Cells were plated onto selective super optimal broth (SOB-streptinomycin) agar and grown overnight. A single colony was inoculated into selective SOB media for overnight growth at 37 °C. After 12 h, cells were diluted into fresh media (500 mL, SOB-strep) and incubated at 37 °C to mid-log phase growth (OD_600_ ∼ 0.9). Protein expression was induced by the addition of IPTG (1 mM final concentration). Cells were maintained at 37 °C for 4 h and then harvested by centrifugation (6k rpm, 10 min). Cell pellets were resuspended in 0.1 M potassium phosphate buffer (pH 7.4), lysed via probe sonication (10 min lysis, 75 W, 5 s intervals) in an ice bath, and centrifuged to remove cellular debris (14k rpm, 10 min). VLPs were precipitated from the clarified cell lysate by the addition of 27.5% ammonium sulfate and incubation with rotation (1 h, 4 °C) followed by centrifugation (14k rpm, 10 min). The resulting protein pellet was resuspended in 0.1 M potassium phosphate buffer and treated with one volume of 1:1/*n*-BuOH/CHCl_3_ to extract water-soluble protein from lipids and aggregates. Following centrifugation (14k rpm, 10 min), the aqueous protein-containing layer was collected and loaded onto 10–40% sucrose density gradients (28k rpm, 4 h), for further purification. VLP bands were isolated via a syringe and pelleted by ultracentrifugation (68k rpm, 2 h). VLP pellets were resuspended in 1× phosphate-buffered saline (PBS), sterilized via 0.2 μm syringe filters, and subsequently characterized.

### Characterization of Qβ VLPs

Protein concentration was determined by Bradford assay (Pierce, Coomassie Plus) against BSA standards. Particles were characterized by FPLC (Superose 6 size exclusion) to determine particle purity and aggregation, and by dynamic light scattering (Wyatt Dynapro plate reader) to determine hydrodynamic radius. The extent of particle modification with azido groups was determined by high-resolution electrospray ionization time-of-flight mass spectrometry (ESI-TOF MS), and the extent of particle modification with oligonucleotides by microfluidic gel electrophoresis (Agilent Bioanalyzer, Protein 80).

### VLP Bioconjugation

**Figure 1:**
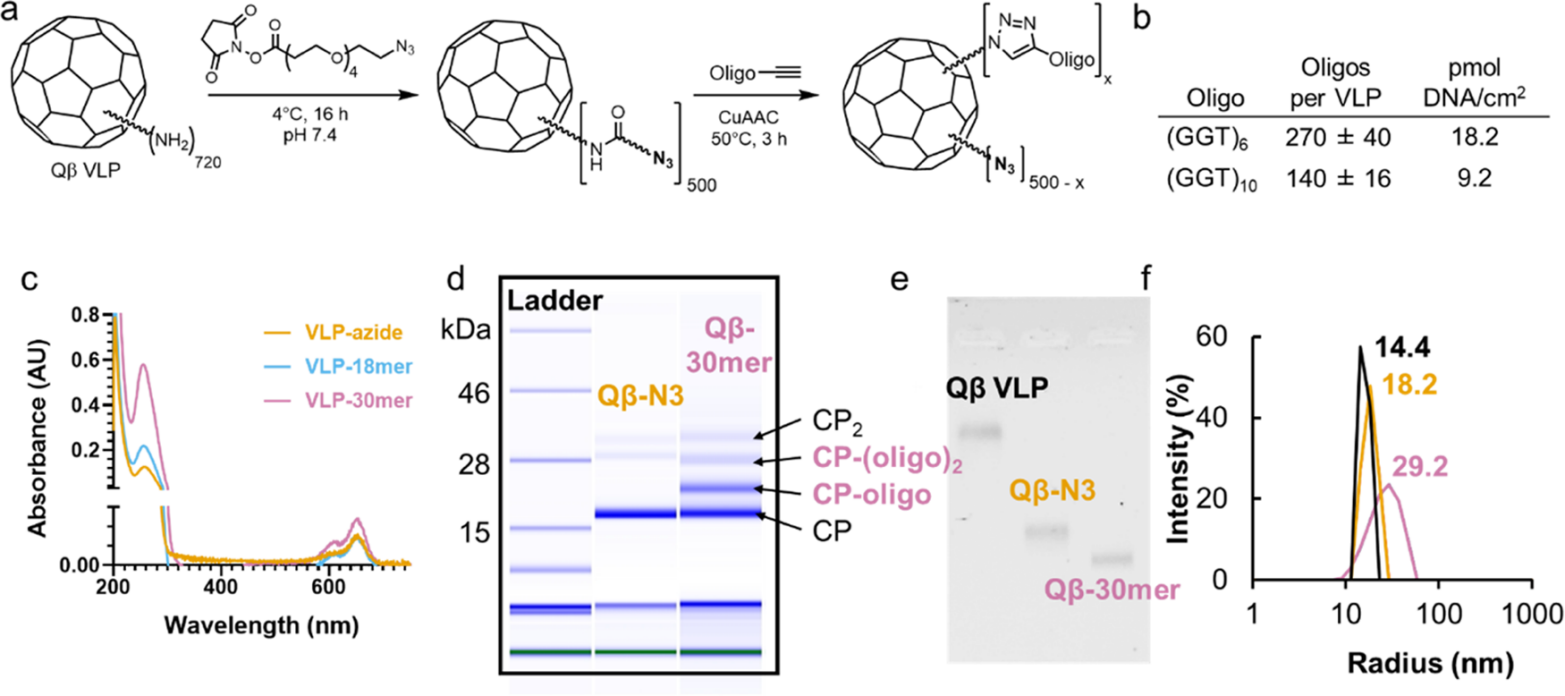
Bioconjugation of oligonucleotides to Qβ nanoparticles. (a) Synthesis of oligo-modified Qβ conjugates. “CuAAC” = CuSO_4_, THPTA, sodium ascorbate, aminoguanidine, 0.1 M potassium phosphate buffer, pH 7.4. Particles were purified by desalting and centrifugal ultrafiltration after the second and third steps. (b) Average calculated density of conjugated oligonucleotides, approximating the VLP as a spherical particle; ^†^density at inner surface, using a radius of 14 nm. (c) UV–vis spectroscopy of native VLP conjugates and (d) microfluidic chip-based electrophoretic mobility analysis of denatured VLP conjugates were used to determine the relative quantitation of modified and unmodified subunits. Analysis of native unmodified (black), azide-labeled (gold), or 30-mer-labeled (pink) particles by (e) nondenaturing agarose gel electrophoresis and (f) dynamic light scattering, resulting in the average hydrodynamic radius noted for each particle.

Amine-reactive succinimidyl ester chemistry was used to install both dye and azide (Figure 1a), followed by oligonucleotide-alkyne ligation *via* copper-catalyzed azide–alkyne cycloaddition. In detail, AlexaFluor647 NHS ester (0.1 μmol) was added to a solution of Qβ VLPs (2 mg, 0.14 μmol in capsid protein subunit) and, after 2 h of gentle mixing at 4 °C, NHS-PEG_4_-azide (21 μmol) was added. The reaction was allowed to proceed overnight at 4 °C with rotation. The reaction mixture was purified using a PD-10 desalting column, followed by centrifugal filtration using an Amicon Ultra 100k MW cutoff device. The extent of particle modification was determined *via* ESI-TOF high-resolution MS (HRMS). The resulting azide-modified particles were treated with alkyne-terminated oligonucleotides in the presence of copper sulfate, tris((1-benzyl-4-triazolyl)methyl)amine (THPTA) or other copper-binding ligands (Table 3), aminoguanidine, and sodium ascorbate as previously described.^59^ Oligonucleotide loading was controlled by the addition of different concentrations of the alkyne (Table 3). Upon completion, reactions were purified by PD-10 desalting columns, followed by centrifugal filtration using an Amicon Ultra 100k *M*_W_ cutoff device, and protein recovery was determined via a Bradford assay. The extent of particle modification with oligonucleotides was determined by microfluidic gel electrophoresis (Protein 80 Kit, Agilent). Particles were characterized as described above to determine particle stability and purity.

**Table 3:**
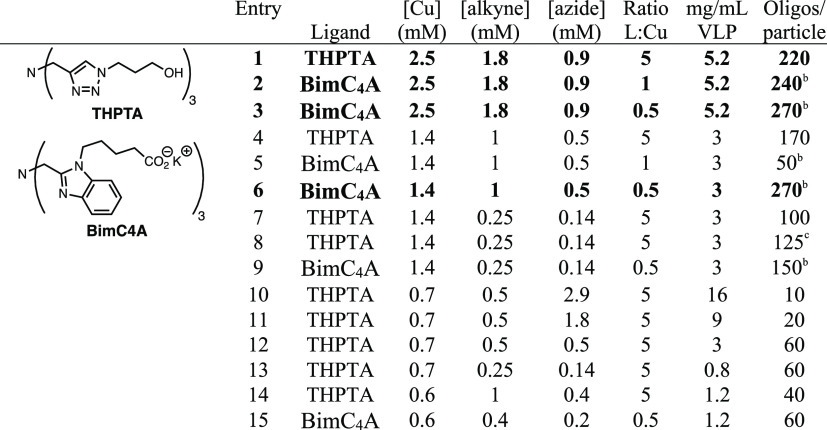
On-VLP CuAAC, Promoted by Copper-Binding Ligands THPTA or BimC_4_A^a^

a Reactions were performed at 50 °C, for 3 h, then analyzed by on-chip electrophoresis.

b Copper/ligand aggregation observed.

c Reactions spiked with additional equivalent of each of copper, ligand, and ascorbate after 1 h.

### Cell Culture

All culture media were purchased from Life Technologies (Carlsbad, CA) unless noted otherwise. HeLa or C166 cells were cultured in supplemented DMEM (10% FBS, 1× sodium pyruvate, 1× GlutaMax, 1× Pen-Strep) and maintained in a humidified incubator (37 °C, 5% CO_2_). The cells were routinely cultured in phenol red containing DMEM but were switched to OptiMEM for all imaging experiments.

### Confocal Microscopy

Cells were cultured at a density of 2.5 × 10^5^ cells/well on glass-bottom Ibidi μ-Slide 8-well plates. After 24 h, the culture media was aspirated and the cells were washed once with 1× PBS, then AF647-labeled VLPs in OptiMEM were added (25 nM VLP final concentration). The cells were incubated with particles in a humidified incubator (37 °C, 5% CO_2_) for 120 min, then washed with 1× PBS (2×), and stained with 10 μg/mL Hoescht 33342 in 1× PBS for 5 min. The cells were imaged immediately in OptiMEM on a Nikon CSU-W1 spinning disk confocal microscope.

### Cell-Surface Oligonucleotide Labeling

Cells were cultured overnight as described above and washed with 1× PBS to remove overnight medium. The cells were then treated with a 1 μM or a 5 μM solution of 5′-cholesterol-terminated oligonucleotide in PBS for 15 min, and then washed with 1× PBS. The remaining steps were as described above.

### Image Analysis

Image analysis was performed in FIJI. Cells were manually selected by freeform, and the integrated density was calculated for each cell. Fluorescence per cell was adjusted by subtracting the area-corrected background for each selection.

### Animal Handling

All animal studies were performed in compliance with the Georgia Institute of Technology Institutional Animal Care and Use Committee. CD-1 IGS mice or BALB/c mice were obtained from Charles River Laboratories (Wilmington, MA) and housed in the Physiological Research Laboratory (PRL) at Georgia Institute of Technology.

### Biodistribution

Six-week-old CD-1 IGS mice (*n* = 3 per VLP group) were obtained from Charles River Laboratories and placed on an alfalfa-free diet. The mice were injected intravenously with either 30 μg of FNIR VLPs or PBS controls on day 0 (0.1 mL per dose in 1× PBS). At the indicated time points (Figure 3), the mice were placed under anesthesia and whole-animal images were acquired using an IVIS SpectrumCT (PerkinElmer) imaging cabinet (745 ex/800 em). Reference images from mice immunized with PBS were used to establish detection thresholds and to adjust for background fluorescence.

### Assessment of Particle Antigenicity

Seven-week-old female BALB/c mice (Charles River Laboratories) were immunized subcutaneously with 20 μg of VLPs or V-SNAs (0.1 mL per dose in 1× PBS), followed by boost inoculations on days 14 and 28. Blood was collected via submandibular bleed on days 0 (immediately prior to immunization), 14, 21, 28, 35, and 42; serum was prepared by centrifugation, and stored at −80 °C until required for analysis. Antiparticle recognition by serum antibodies was monitored by ELISA, as follows: VLPs (**1** μg/mL in 1× PBS) were plated on high-binding plates and incubated overnight at 4 °C. Plates were washed thrice with PBST (1× dPBS ca. 0.05% Tween-20) to remove unbound protein, incubated in casein blocking buffer (G Biosciences#097B) on a rotary incubator (2 h, room temperature), and washed once again (PBST). Serial dilutions of sera from immunized mice at six serial dilutions (from 1:125 to 1:512,000) in blocking buffer were plated and incubated on a rotary incubator (1 h, room temperature). The plates were washed to remove unbound serum antibodies and were incubated with a secondary reporter goat anti-mouse IgG HRP (Southern Biotech) at 1:2500 dilution in blocking buffer on a rotary shaker (1 h, room temperature); for subclass ELISAs, the appropriate secondary reporters were used instead. Plates were washed with PBST and developed by adding 1-step Ultra TMB (Fisher Scientific) for 60 s, followed by quenching with 2 M H_2_SO_4_. Absorbance (450 nm) was measured by a plate reader (Varioskan Flash, Thermo Fisher) and titers were calculated by sigmoidal nonlinear regression using GraphPad Prism.

## Results and Discussion

### Analysis of CuAAC Ligands for Oligonucleotide Conjugation

Copper(I)-catalyzed azide–alkyne cycloaddition (CuAAC) benefits dramatically from ligand-accelerated catalysis,^34^ but the nature of the optimal ligand must be chosen with the reaction conditions in mind, primarily the presence of potential Cu-binding agents such as solvent, certain amino acid residues, and other additives.^35,36^ While tris(hydroxypropyltriazolylmethyl)amine (THPTA) or analogous tris(triazolylmethyl) structures are recommended for most aqueous-phase bioconjugation applications,^37^ creating a dense array of oligonucleotides is an unusual goal, one that creates significant changes in the molecular microenvironment as the reaction proceeds. Since it was therefore not certain that THPTA would be the ligand of choice in this case, we also tested two versions of a tris(benzimidazole)-style ligand, which binds more tightly to Cu ions, one bearing pendant carboxylic ester groups (BimC_4_E) and the other carboxylic acids (BimC_4_A) (Table 2).^35^

An initial test of solution-phase reactivity was performed using the fluorogenic reaction of a 30-mer oligonucleotide (GGT)_10_, alkynylated by the reaction of C3-amino-labeled oligonucleotides with an alkyne-terminated pentynoic *N*-hydroxysuccinimidyl ester, with an excess of coumarin azide reporter, each compared to the signal generated by the standard reaction of propargyl alcohol with coumarin azide catalyzed by Cu-THPTA (Table 2). Under these conditions, THPTA outperformed both benzimidazole ligands, with the best reactivity approaching that of the reference reaction employing a small-molecule alkyne (entry 4). The acid-functionalized BimC_4_A performed marginally better than the ester-containing ligand. Each ligand catalyzed the reaction best when used in an 0.5:1 ratio with copper, at the cost of sacrificing some oxidative protection that these copper-binding ligands provide during CuAAC (Figure S2).^37^ Still, THPTA and BimC_4_A, but not BimC_4_E, showed good protection of oligonucleotides from copper-mediated degradation (Figure S2). The poor outcome with BimC_4_E may be due to its lower aqueous solubility compared to the other ligands, requiring its use at lower concentrations. Oligonucleotides were further protected during CuAAC by the addition of aminoguanidine, equimolar with sodium ascorbate, at the cost of some reaction efficiency (Figure S4).^37^ Consistent with previous observations,^34,36,37^ THPTA ligand was much less inhibitory than the other ligands when used in excess (entries 4 vs 1; 5 vs 2), and the reaction was strongly sensitive to overall concentration (entry 4 vs 10).

The relative efficiencies of THPTA and BimC_4_A in promoting polyvalent CuAAC conjugation to VLP scaffolds were explored with alkyne-labeled 18-mer (GGT)_6_ or 30-mer (GGT)_10_ oligonucleotides and azide-labeled VLPs; these sequences were selected because of previously demonstrated use in cell uptake of DNA-decorated scaffolds.^11,26,38^ These particles were created by acylating surface amines with an NHS-tetraglyme-azide linker (Figure 1a). The CuAAC reactions were performed at high concentrations of the Cu–ligand complexes (0.6–2.5 mM in copper ions) and at an elevated temperature (50 °C) for 3 h to enhance reactivity in the challenging task of stapling high densities of oligonucleotides to the nanoparticle scaffold. The extent of reaction in each case was determined by on-chip electrophoresis of denatured samples and quantification by integration of the band intensities corresponding to modified and unmodified subunits. Use of the (BimC_4_A)_3_ ligand yielded conjugates with somewhat higher densities of oligonucleotides, reaching average maxima of ∼140 copies of the 30-mer or ∼270 copies of the 18-mer oligonucleotides per VLP, respectively (Figure 1a–d). Standard characterization techniques showed the resulting V-SNAs to be intact (size exclusion chromatography) and well controlled in size (dynamic light scattering). The combination of the azido-PEG linker and the surface-displayed oligonucleotides is expected to make the particles more negatively charged (Figure 1e) and nearly double the average hydrodynamic radii of the particles (Figure 1f).

When combined with a dsDNA crosslinker that contained ssDNA overhangs complementary to particle-displayed sequences, V-SNAs could be assembled into larger structures, with greater apparent retention time via native agarose gel electrophoresis and a larger radius by light scattering (Figure S6). Treatment of the resulting particles with a high concentration of DNase I (0.1 mg/mL) reversed apparent VLP crosslinking (Figure S7).

### Oligonucleotide Conjugation Inhibits Immune Recognition of VLPs

Because VLPs traffic predominantly to the liver, we sought to compare immunogenicity, liver localization, and cellular uptake properties of densely labeled V-SNAs relative to unmodified particles. The choice of GGT repeats was motivated by the reported role of hydrophilic polymers^39,40^ in reducing the immunogenicity of carrier proteins and by the ability of GGT repeat sequences to form a G-quadruplex shell structure when attached to SNAs,^26^ suggesting that these molecules may protect VLP scaffolds from immune recognition.

**Figure 2:**
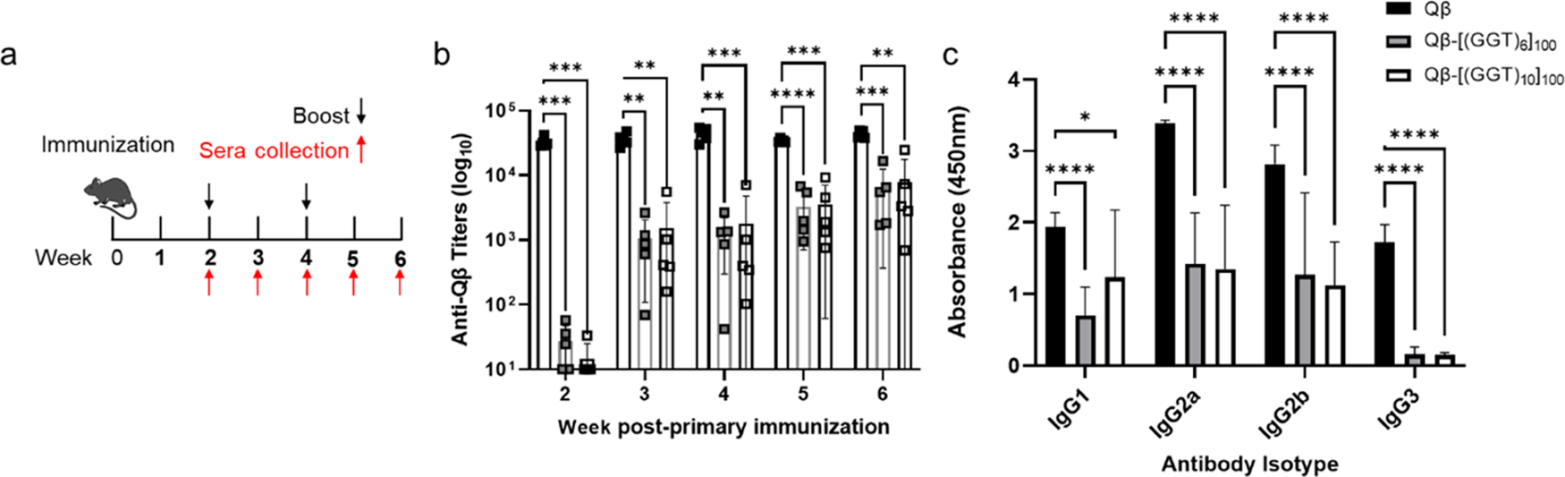
In vivo analysis of particle immunogenicity. (a) Immunization schedule; all doses 20 μg VLP; 5 mice per group. (b) Antiparticle IgG titers from mice immunized with indicated particles. Titer curves were analyzed using nonlinear regression curve fitting in Prism, and the titer was calculated as the midpoint of the corresponding curve fit; symbols indicate the mean value for each group ± standard error. (c) IgG subclass distribution from week 5 sera against indicated WT or oligo-modified particles. Symbols indicate the mean value for each group ± standard error. Statistical analysis performed using a one-way ANOVA with Šídák’s multiple comparisons test. **** = *p* ≤ 0.0001; *** = *p* ≤ 0.001; ** = *p* ≤ 0.01; * = *p* ≤ 0.05.

BALB/c mice were immunized subcutaneously with either V-SNAs or unmodified VLPs, and anti-Qβ IgG in serum was monitored over time (Figure 2a). Compared to sera from mice immunized with unmodified particles, sera from mice immunized with oligonucleotide-functionalized particles consistently gave significantly lower overall antiprotein IgG titers against plated Qβ-VLPs (Figure 2b). Lower absolute titers against all IgG subclasses were also observed, as well as a lower relative IgG3 response (Figure 2c); these observations are consistent with reduced antigenicity of V-SNAs.

### Liver Retention Time

**Figure 3:**
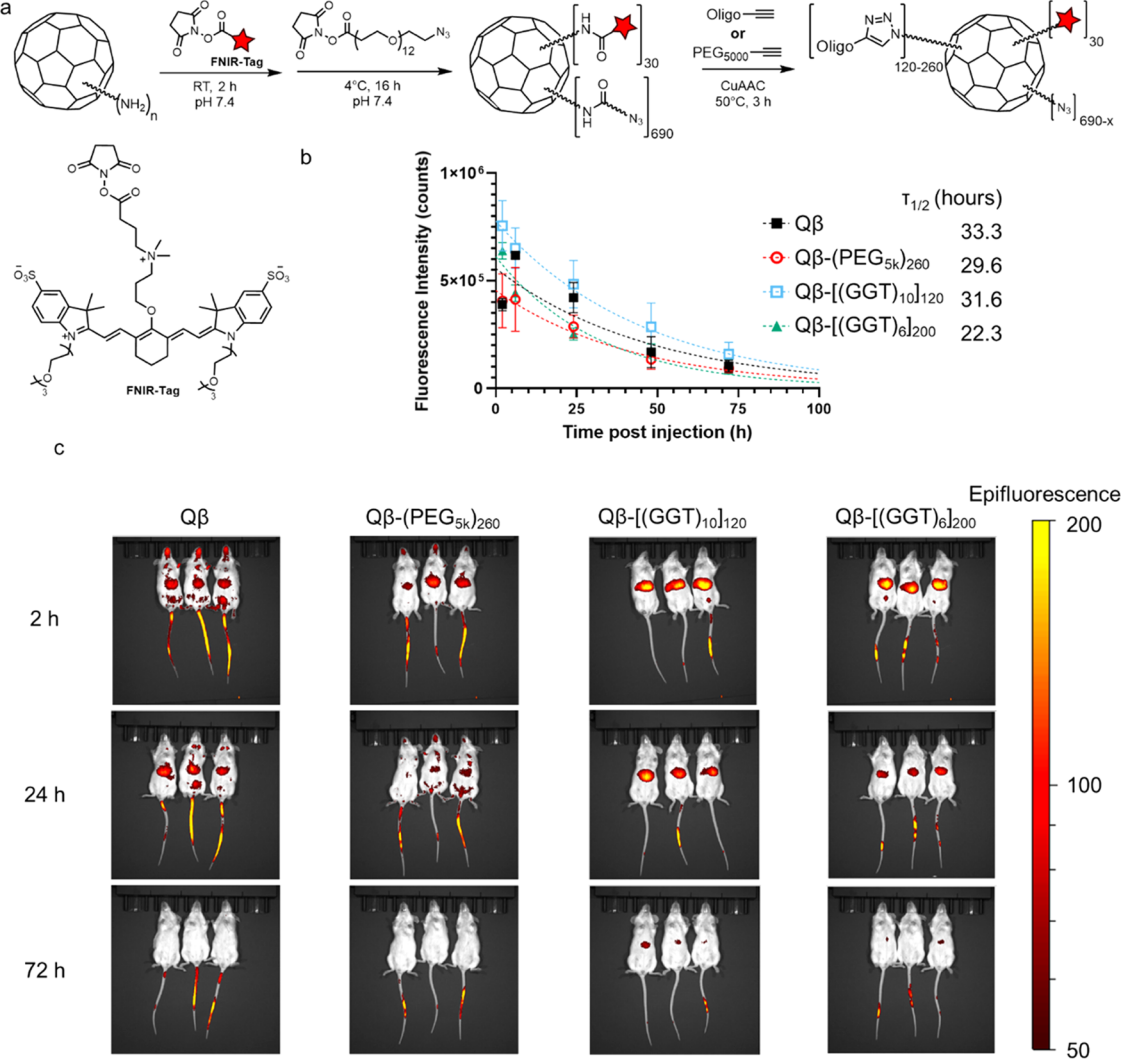
Liver retention of Qβ-DNA conjugates. (a) Preparation of FNIR-Tag-labeled particles. (b) Fluorescence intensity quantitation of liver-associated VLPs from whole-animal IVIS imaging. Data were fit to a one-phase decay nonlinear regression in GraphPad Prism. Error bars are the SEM from biological replicates (*n* = 3).

Virus-like particles and other nanoparticles of similar size are cleared from systemic circulation by the liver, usually rapidly.^41−45^ However, subsequent clearance from the liver, presumably by proteolytic breakdown and biliary excretion, is not often characterized. This parameter can be important for functional outcomes of certain nanoparticle agents proposed to be active in the liver, such as viral vectors for gene delivery or particulate agents for immunological modulation. We determined how long the particles remain detectable in the liver by first functionalizing the capsid with a small number of zwitterionic near-IR fluorophores,^44^ followed by dense labeling with either an oligonucleotide [(GGT)_6_, (GGT)_10_] or a PEG_5000_ shield (Figure 3a). A dose of 30 μg of each VLP conjugate was administered to CD-1 mice by tail vein injection and gross organ distribution assessed by whole-animal fluorescence imaging to monitor liver retention and clearance of particles (Figures 3b and S8). No dramatic differences were observed: particles decorated with either PEG or the longer oligonucleotide were found to be visible in the liver for a similar period (half-life = 29.6 and 31.6 h, respectively) as unmodified particles (half-life = 33.3 h), whereas particles bearing a higher density of shorter oligonucleotides were cleared from the liver somewhat more rapidly (half-life = 22.3 h).

### Delivery of V-SNAs In Vitro

**Figure 4:**
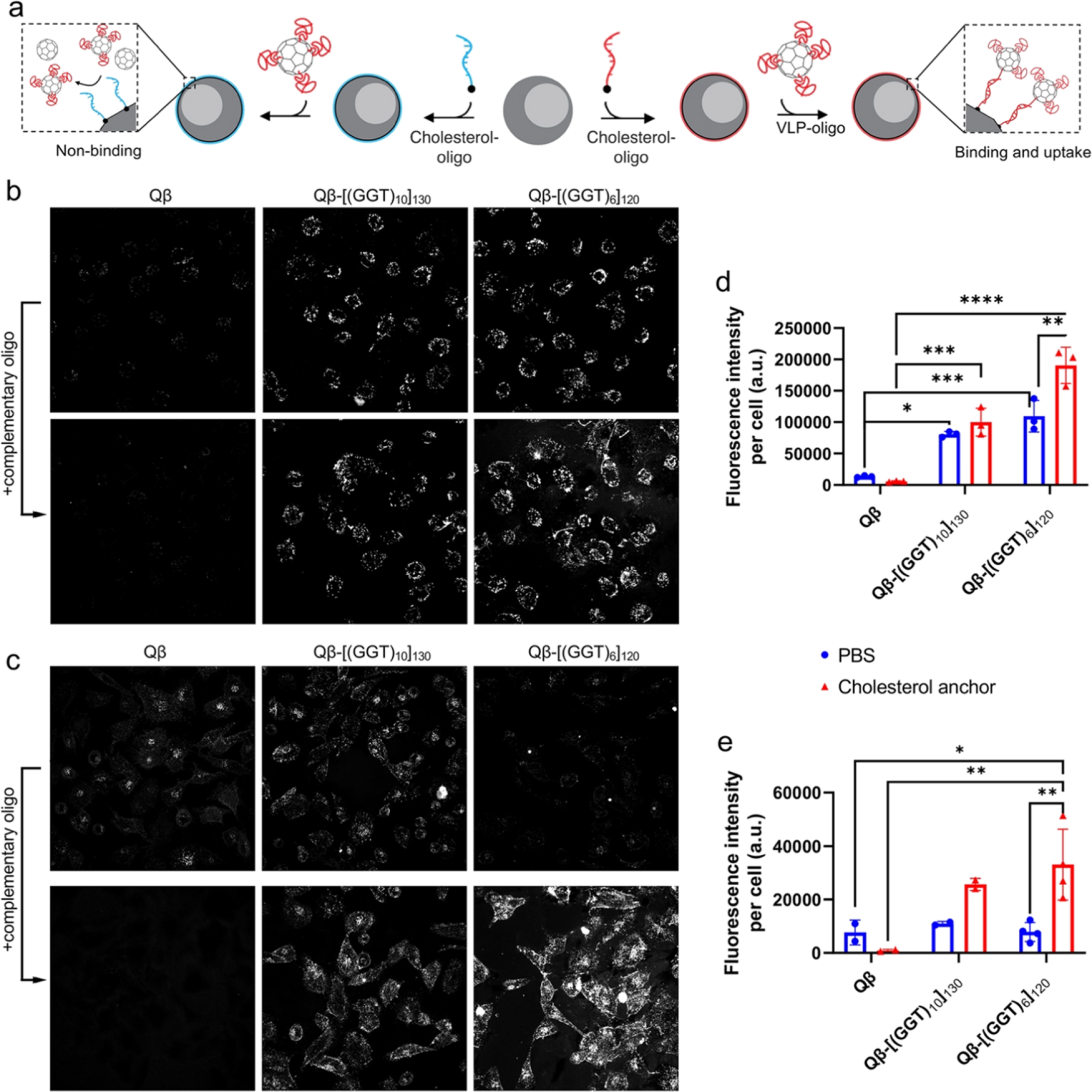
Representative live-cell spinning disk confocal microscopy images. (a) Schematic demonstrating cholesterol–oligonucleotide insertion into cell membranes and subsequent impact on VLP internalization. (b) C166 or (c) HeLa cells were dosed with 5 nM Qβ-VLPs for 180 min or pretreated with 5 μM cholesterol-terminated anchor oligonucleotides for 15 min and washed prior to treatment with 5 nM Qβ VLPs for 180 min. Maximum intensity projections for *z*-stack confocal images are shown in (b) and (c). (d, e) Mean fluorescence intensity per cell from data such as in (b) and (c), respectively. Statistics were performed in Prism by two-way ANOVA with Tukey’s multiple comparison test; **** = *p* ≤ 0.0001; *** = *p* ≤ 0.001; ** = *p* ≤ 0.01; * = *p* ≤ 0.05.

We also measured V-SNA conjugate uptake in cultured HeLa and C166, epithelial and endothelial cell lines, respectively, typically used to assess the uptake of SNAs.^26^ Fluorescent V-SNAs were prepared, bearing an average of 30–40 AlexaFluor647 dyes attached via amine acylation, and ca. 100–130 15-mer [(GGT)_5_] or 30-mer [(GGT)_10_] oligonucleotides attached via CuAAC, as above. As negative controls, and as examples of particles with nonspecific cell-binding properties,^46^ we used VLPs labeled with fluorescent dyes, with no further modification. Using confocal microscopy, we found that V-SNAs were bound and internalized by C166 cells to a greater extent than the unmodified control particles (Figure 4a,c), while V-SNAs and control particles were captured similarly by HeLa cells (Figure 4b,d). In nearly all cases, we observed punctate staining throughout the cell, suggesting dominant endosomal or vesicular uptake, in contrast to the type of diffuse pattern that would be observed for simple cell-surface binding (Figure S9 and Supporting Movies S1–S3). With HeLa cells, we observed a mixture of punctate staining and cell-surface binding for control particles, but not V-SNAs, suggesting that, although similar levels of particles are captured by HeLa, V-SNAs are preferentially internalized over control particles (Figure S10 and Supporting Movies S4–S6).

**Figure 5:**
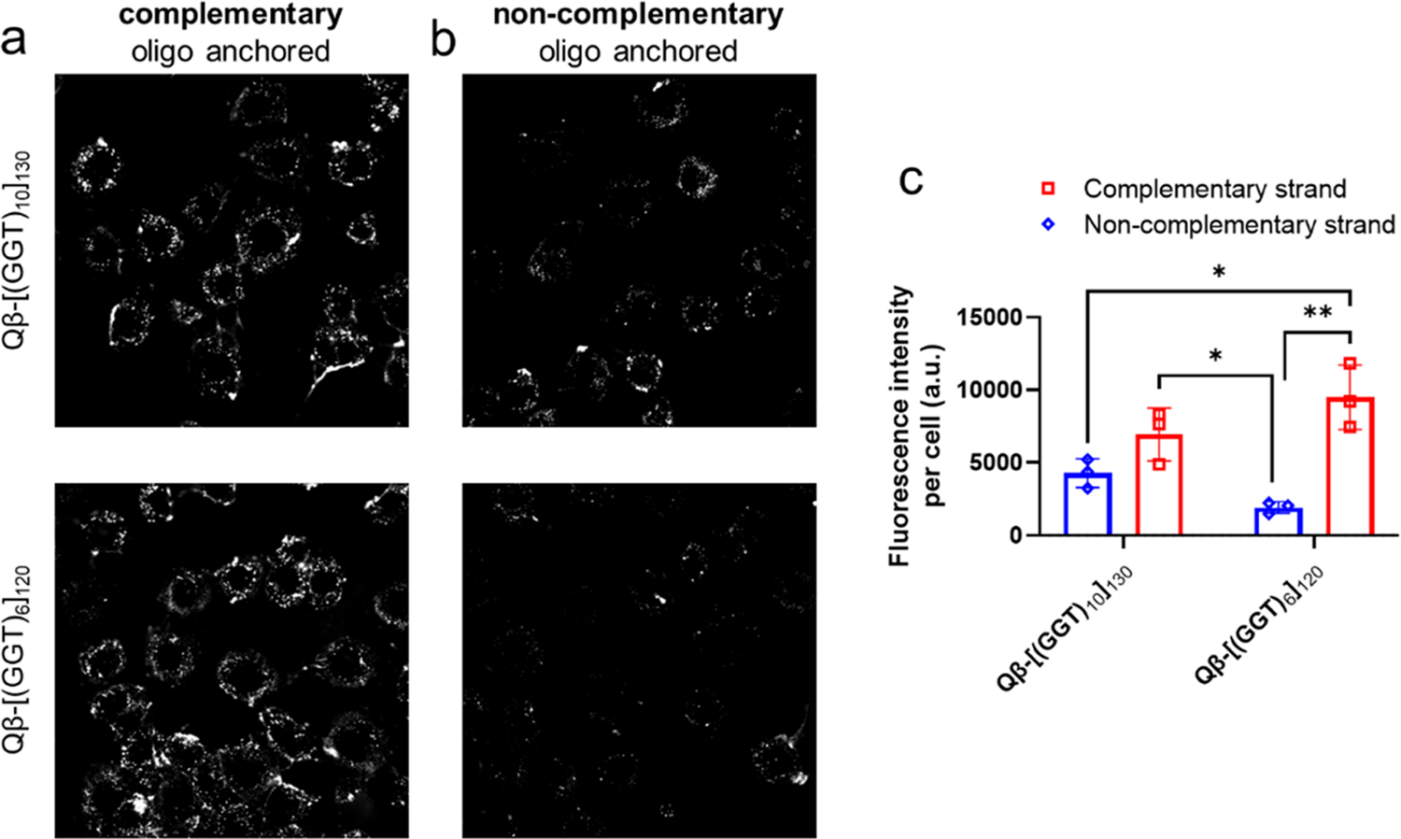
Comparison of cell-surface anchoring of DNA complementary or noncomplementary to VLP-displayed DNA. C166 cells pretreated with 1 μM cholesterol-anchored oligonucleotides for 15 min, washed in PBS, and treated with 2.5 nM VLP-18-mer or VLP-30-mer for 90 min. Cell-anchored oligonucleotides were (a) complementary or (b) noncomplementary to VLP-displayed strands. (c) Mean fluorescence intensity per cell from data such as in (a) and (b). Statistics were performed in Prism by two-way ANOVA with Tukey’s multiple comparison test; ** = *p* ≤ 0.01; * = *p* ≤ 0.05.

It is well established that lipid-terminated oligonucleotides can insert into cell membranes and enable cellular adhesion via sequence-specific hybridization,^47,48^ so we used this strategy to enhance uptake of V-SNAs. For both HeLa and C166, pretreatment with a single-stranded cholesterol-labeled oligonucleotide [cholesterol-(CCA)_5_] significantly increased the extent of punctate staining by dye-labeled VLPs bearing complementary (GGT)_6_ sequences, but not the longer (GGT)_10_ oligonucleotides. This phenomenon was also sequence-dependent: cells pretreated with a noncomplementary cholesterol–oligonucleotide conjugate demonstrated diminished uptake of V-SNAs (Figure 5), with a greater differential effect again observed with 18-mer-labeled VLPs compared to the display of 30-mers. Furthermore, the uptake of unmodified VLPs by both HeLa and C166 cells was diminished by pretreatment of cells with cholesterol-terminated oligonucleotides (Figure 4b,c).

**Figure 6:**
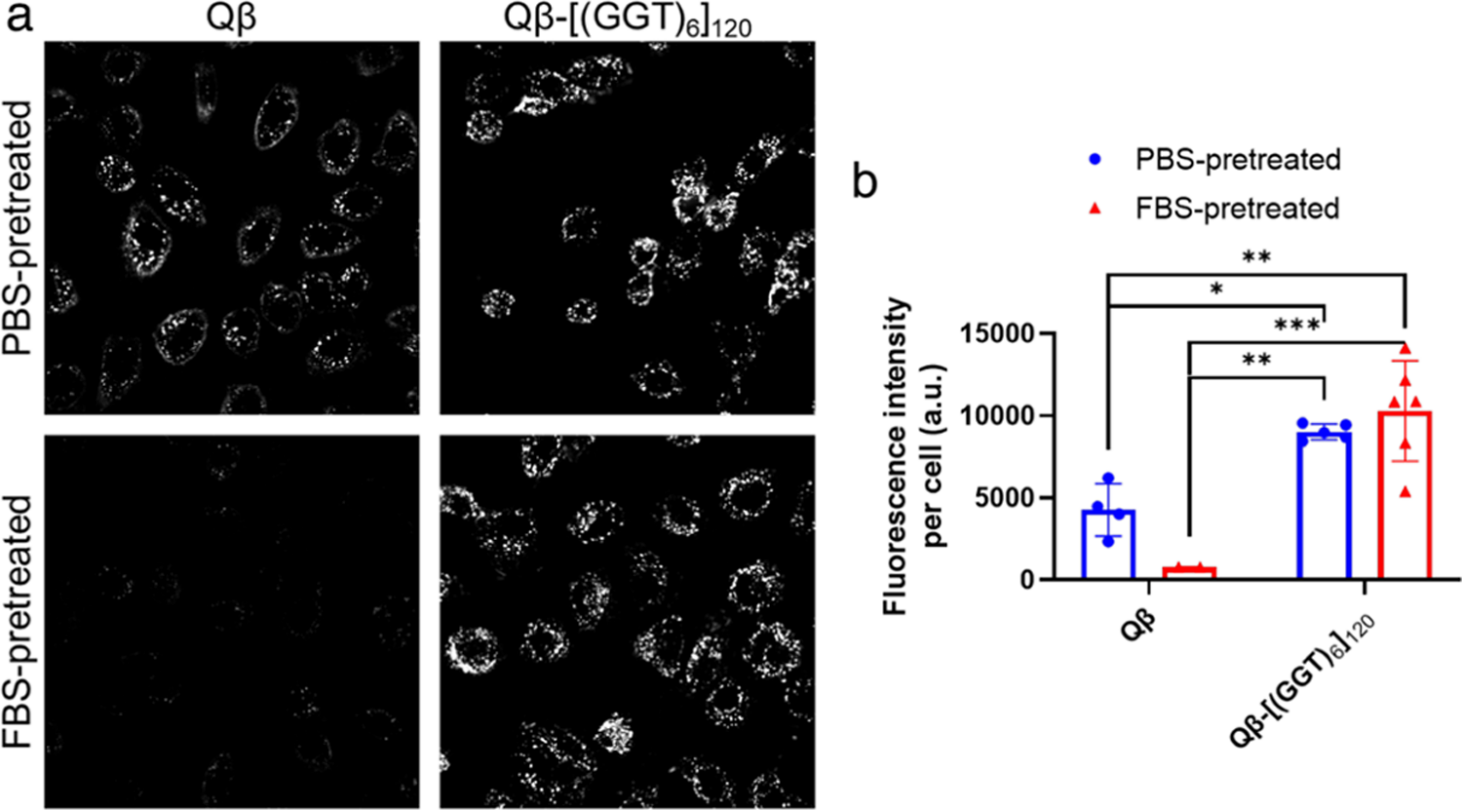
Representative live-cell spinning disk confocal microscopy images showing that V-SNAs retain enhanced cellular uptake following prolonged incubation in FBS. (a) Qβ-VLPs were pretreated with (top) 1xPBS or (bottom) 20% FBS for 16 h at 37 °C, after which C166 cells were dosed with 5 nM VLPs diluted in OptiMEM for 90 min. (b) Mean fluorescence intensity per cell from data such as in (a). Statistics were performed in Prism by two-way ANOVA with Tukey’s multiple comparison test; *** = *p* ≤ 0.001; ** = *p* ≤ 0.01; * = *p* ≤ 0.05.

To assess the functional stability of VLPs to prolonged incubation in serum, we incubated either V-SNAs or control VLPs in 20% FBS overnight at 37° C and then measured the relative uptake of particles (Figure 6). V-SNAs retained their relatively greater uptake properties when treated with serum-containing media, while underivatized VLPs incubated in serum were bound to a much lesser extent. The corresponding single-stranded oligonucleotide was significantly degraded within the same timeframe. These data suggest that V-SNAs are resistant to serum-mediated nuclease degradation of the conjugated oligonucleotides and that V-SNAs may prevent serum-induced uptake inhibition by inhibiting the formation of a protein corona.

## Conclusions

The CuAAC is a potent bioconjugation reaction, but the potential for Cu(I)-associated oxidative damage to biomolecules can limit its use in sensitive applications. We routinely remove Cu ions from bioconjugation products by dialysis against EDTA or by incubation with Cu-trapping resins such as Cuprisorb (note that these resins can trap some proteins); such procedures reduce Cu concentrations to below 1 ppm and we have never detected Cu-related toxicity in many studies using such materials. In the present work, we screened known copper-binding ligands for their ability to accelerate click reactions on oligonucleotide substrates without causing undesirable damage and recommend the Bim(C_4_A)_3_ ligand as somewhat superior to THPTA for the creation of highly dense presentations of DNA, a revealing finding given that THPTA performed better in test reactions of oligonucleotides with a small-molecule azide.

The reasons for this difference in performance have not been explored, but are related to (a) the nature of the microenvironment at the reaction sites as oligonucleotides are attached, becoming unusually high in ionic strength, and (b) the presence of carboxylate groups, rather than esters, on the ligand since Bim(C_4_E)_3_ is significantly less effective. We suggest two possible hypotheses which are difficult to distinguish. First, it is possible that the carboxylic acids or carboxylate anions of Bim(C_4_A)_3_ mediate favorable interactions with the phosphate anions or cationic counterions of the developing layer of covalently attached oligonucleotides, thereby increasing the concentration of catalyst at the VLP surface. Alternatively, if copper–phosphate binding is deleterious to the CuAAC reaction, the pendant carboxylates of the ligand may engage in stabilizing interactions with the metal center, outcompeting phosphate.

Mirkin and co-workers have suggested that many biological properties of nanoparticles can be manipulated by the selection of different linkers and displayed oligonucleotide sequences.^26^ Although it is well established that oligonucleotide density on SNAs tends to correlate with cellular uptake^49^ and nuclease resistance,^50,51^ other functions, such as immune stimulation^52,53^ or gene silencing,^54,55^ do not necessarily directly depend on nucleic acid density alone. Other factors, such as platform stability, biodistribution, and function of the attached oligonucleotides, are linked to potency, as was nicely demonstrated in a recent high-throughput screening of liposomal SNAs.^56^ The studies described here represent a starting point for the exploration of these questions using virus-like particle platforms, focusing on oligonucleotide length and attachment density. We employed the GGT repeat motif because ProSNAs bearing GGT repeats in this fashion are more efficiently captured than SNAs bearing other oligonucleotides.^26^

The resulting structures, called V-SNAs in analogy to nomenclature for other nanoparticles, exhibited properties that differ from unfunctionalized virus-like particles. Protein antigenicity was found to be significantly suppressed while cellular uptake in cultured cells was enhanced. Uptake occurred to a significantly greater degree for C166 cells, a murine endothelial cell line expressing VCAM-1 and used for differentiation, compared to HeLa cells. Surface labeling of cell membranes with complementary oligonucleotides greatly increased V-SNA binding and uptake. This phenomenon showed an interesting dependence on oligonucleotide length, being more efficient for VLPs displaying the 18-mer sequence than the 30-mer repeat, in each case presented with a complementary cell surface-displayed 15-mer. This contrasts with a previous report that SNAs bearing a 30-mer GGT repeat were better internalized by C166 than SNAs bearing shorter GGT repeat sequences [(GGT)_2_T_24_, (GGT)_4_T_18_, (GGT)_6_T_12_, or (GGT)_8_T_6_], noting that a threshold around 4–6 GGT repeats enabled more efficient capture.^38^ Since the densities of 18- and 30-mer attachment to Qβ were very similar, we speculate that the particle-displayed 30-mer repeat sequence forms a more stable G-quadruplex secondary structure, making the 18-mer repeat sequence more sensitive to capture by cells bearing a complementary strand.

Prolonged incubation in serum reduced the cellular uptake of unmodified particles but did not significantly impact V-SNA uptake, suggesting that the oligonucleotide-functionalized particles are resistant to serum nuclease degradation and serum protein adsorption. Retention of particles in the liver was only modestly affected by DNA conjugation, and that for only the shorter of the DNA sequences tested. Thus, high-density DNA attachment may be regarded as a promising strategy for modulating the properties of virus-like particles, as it has for other nanoparticle scaffolds.

## Supporting Information Available

Experimental details and additional data regarding CuAAC reactions; biodistribution, liver clearance, and intracellular localization, including six movies showing *z*-stacked images of cells treated with dye-labeled particles (PDF)
*Z*-stack image series of C166 cells treated with 5 nM Qb-VLPs for 180 min, followed by washing with PBS and treatment with LysoTracker (100 nM) and Hoescht (10 μg/mL), image stacks show signal from nuclear (cyan), lysosomal (yellow), and VLP (magenta) staining (AVI)
*Z*-stack image series of C166 cells treated with 5 nM Qb-VLPs for 180 min, followed by washing with PBS and treatment with LysoTracker (100 nM) and Hoescht (10 μg/mL), image stacks show signal from nuclear (cyan), lysosomal (yellow), and VLP (magenta) staining (AVI)
*Z*-stack image series of C166 cells treated with 5 nM Qb-VLPs for 180 min, followed by washing with PBS and treatment with LysoTracker (100 nM) and Hoescht (10 μg/mL), image stacks show signal from nuclear (cyan), lysosomal (yellow), and VLP (magenta) staining (AVI)
*Z*-stack image series of HeLa cells treated with 5 nM Qb-VLPs for 180 min, followed by washing with PBS and treatment with Hoescht (10 μg/mL), image stacks show signal from nuclear (cyan) and VLP (magenta) staining (AVI)
*Z*-stack image series of HeLa cells treated with 5 nM Qb-VLPs for 180 min, followed by washing with PBS and treatment with Hoescht (10 μg/mL), image stacks show signal from nuclear (cyan) and VLP (magenta) staining (AVI)
*Z*-stack image series of HeLa cells treated with 5 nM Qb-VLPs for 180 min, followed by washing with PBS and treatment with Hoescht (10 μg/mL), image stacks show signal from nuclear (cyan) and VLP (magenta) staining (AVI)

## Supplementary Material

bm3c00178_si_001.pdf

bm3c00178_si_002.avi

bm3c00178_si_003.avi

bm3c00178_si_004.avi

bm3c00178_si_005.avi

bm3c00178_si_006.avi

bm3c00178_si_007.avi
