# Supplementary material for: Preparation and Biological Properties of Oligonucleotide-Functionalized Virus-like Particles
Source: Biomacromolecules. 2023 May 31;24(6):2766–76. doi: 10.1021/acs.biomac.3c00178 (PMC10265708; doi:10.1021/acs.biomac.3c00178)
Supplement: Supplementary file 1 — bm3c00178_si_001.pdf [file bm3c00178_si_001.pdf]

Preparation and biological properties of oligonucleotide-functionalized virus-like particles

Robert Hincapie,[a] Sonia Bhattacharya,[a] Parisa Keshavarz-Joud,[a] Asheley Chapman,[a] Stephen N. Crooke,[a] M.G. Finn[a,b,]*

[a]School of Chemistry and Biochemistry, [b]School of Biological Sciences, Georgia Institute of Technology, 901 Atlantic Drive, Atlanta, GA, 30332, USA

* mgfinn@gatech.edu

Supplementary information

General. Reagents and buffers were used as received from the manufacturer, unless otherwise noted.

Virus-like particle preparation and characterization.

Qβ VLPs were expressed and isolated as described in the main text. Particle integrity and homogeneity were assessed through fast protein liquid chromatography (FPLC), performed on an Agilent 1200 using a Superose-6 column (GE/Cytiva), and through dynamic light scattering (DLS), performed on a Dynapro plate reader (Wyatt Technologies). The extent of VLP conjugation was determined through high resolution mass spectrometry, performed on a 6230B time-of-flight LC/MS, using a Poroshell 300SB-C3 LC column (Agilent), and through on-chip electrophoresis (Protein 80 kit) using a 2100 Bioanalyzer (Agilent). Oligonucleotide-amine or oligonucleotide-alkyne reagents were purchased from Integrated DNA technologies (IDT) and used as received.

Qβ-(AF647)$_{30}$(azide)$_{550}$

To a solution of Qβ (1 mL from 8 mg/mL stock solution in 1x PBS, pH 7.4; 0.57 µmol in coat protein (CP) subunit), AFDye 647 NHS ester (16 µL from 25 mM stock solution in DMSO; approx. 0.7 equivalents relative to CP subunit concentration) was slowly added in a microcentrifuge tube. The reaction mixture was wrapped in foil to protect from light and placed on a slowly rotating rotisserie shaker at room temperature (r.t.). After 2 h, azido-PEG$_4$-NHS ester (160 µL from 250 mM stock in DMSO; approx. 70 equivalents relative to CP subunits) was added to the reaction mixture without purification. The reaction was let proceed for another 2 h at room temperature, and the resulting VLPs were purified using PD-10 Sephadex G-25 columns. Eluted particles were concentrated using Amicon Ultra-4 10 kDa centrifugal filters and passed through 0.2 µm PTFE syringe filters. Protein recovery was quantified using the Coomassie Plus Bradford Assay Kit (Pierce). The density of particle-displayed azide linkers was determined by ESI-TOF HRMS (550 ± 50 azides per particle, 3.1 ± 0.3 per subunit), and the density of particle-displayed AF647 dyes was determined by UV-vis spectroscopy. Particle integrity was assessed through DLS and FPLC.

Representative procedure for Qβ-DNA conjugation using THPTA

To a solution of Qβ-(AF647)$_{30}$(azide)$_{500}$ (0.1 mL from 6 mg/mL stock solution in 1x PBS; 38 nmol CP subunit, 106 nmol reactive azides) was added oligonucleotide-alkyne (17 µL from a 10 mM solution in nuclease-free water; 1.6 equivalents relative to particle-azide), a premixed solution of 5:1/THPTA:Cu (8.5 µL from a solution of 450 mM THPTA and 90 mM Cu(II)SO$_4$ in water; 7.2 equivalents copper relative to particle-azide), aminoguanidine (8.5 µL from a 1 M stock in water; approx. 80 equivalents to azide), and sodium ascorbate (8.5 µL from a freshly prepared 1 M stock in water; approx. 80 equivalents to azide). Particles were mixed by gentle pipetting, spun down, and incubated at 50 °C for 3 h in a thermocycler. VLP-oligonucleotide conjugates were purified by using PD-10 Sephadex G-25 column, followed by repeated cycles of centrifugation against Amicon Ultra-4 100 kDa centrifugal filters. Protein recovery was quantified by Bradford assay, as described above. V-SNAs were characterized by UV-Vis spectroscopy

and by microchip electrophoresis using the Protein 80 kit on a 2100 BioAnalyzer (Agilent) to determine the loading density of oligonucleotides on particles, and by DLS and FPLC to determine particle integrity.

Representative procedure for Qβ-(18mer)$_{270}$ conjugation using BimC$_4$A

To a solution of Qβ-(AF647)$_{30}$(azide)$_{500}$ (5 µL from 29 mg/mL stock; 9.2 nmol CP subunit, 25.6 nmol reactive azides) was added oligonucleotide-alkyne (8.6 µL from 6 mM stock; 2.0 equivalents relative to particle-azide), BimC$_4$A (8.4 µL from 4.2 mM stock; 35.3 nmol), $CuSO_4$ (8.4 µL from 8.4 mM stock; 70.6 nmol, 2 equivs relative to ligand), aminoguanidine (2 µL from a 1 M stock in water; approx. 80 equivalents to azide), and sodium ascorbate (2 µL from a freshly prepared 1 M stock in water; approx. 80 equivalents to azide). Particles were mixed by gentle pipetting, spun down, and incubated at 50 °C for 3 h in a thermocycler. Mixtures of the BimC$_4$A ligand and copper in water form precipitates that can be resuspended by dilution to 1-2 mM in water.

Table S1. Oligonucleotides sequences used.

Entry	Purpose	Sequence
1	Fluorogenic or VLP CuAAC (18mer)	Hexynyl-GGTGGTGGTGGTGGTGGT
2	Fluorogenic or VLP CuAAC (30mer)	Hexynyl-GGTGGTGGTGGTGGTGGTGGTGGTGGTGGT
3	VLP crosslinking, strand 1	AAGGAAGGAAGGAAGGAAGGAATTTTTTTTTACCACCA CCACCACCACC
4	VLP crosslinking, strand 2	TTCCTTCCTTCCTTCCTTCCTTTTTTTTTTTACCACCACCA CCACCACC
5	Cell membrane-anchoring (complementary)	CCACCACCACCACCA-TEG-Cholesterol
6	Cell membrane-anchoring (noncomplementary)	ACACGCATCAGCGCACGTAAGCAGC-TEG-Cholesterol

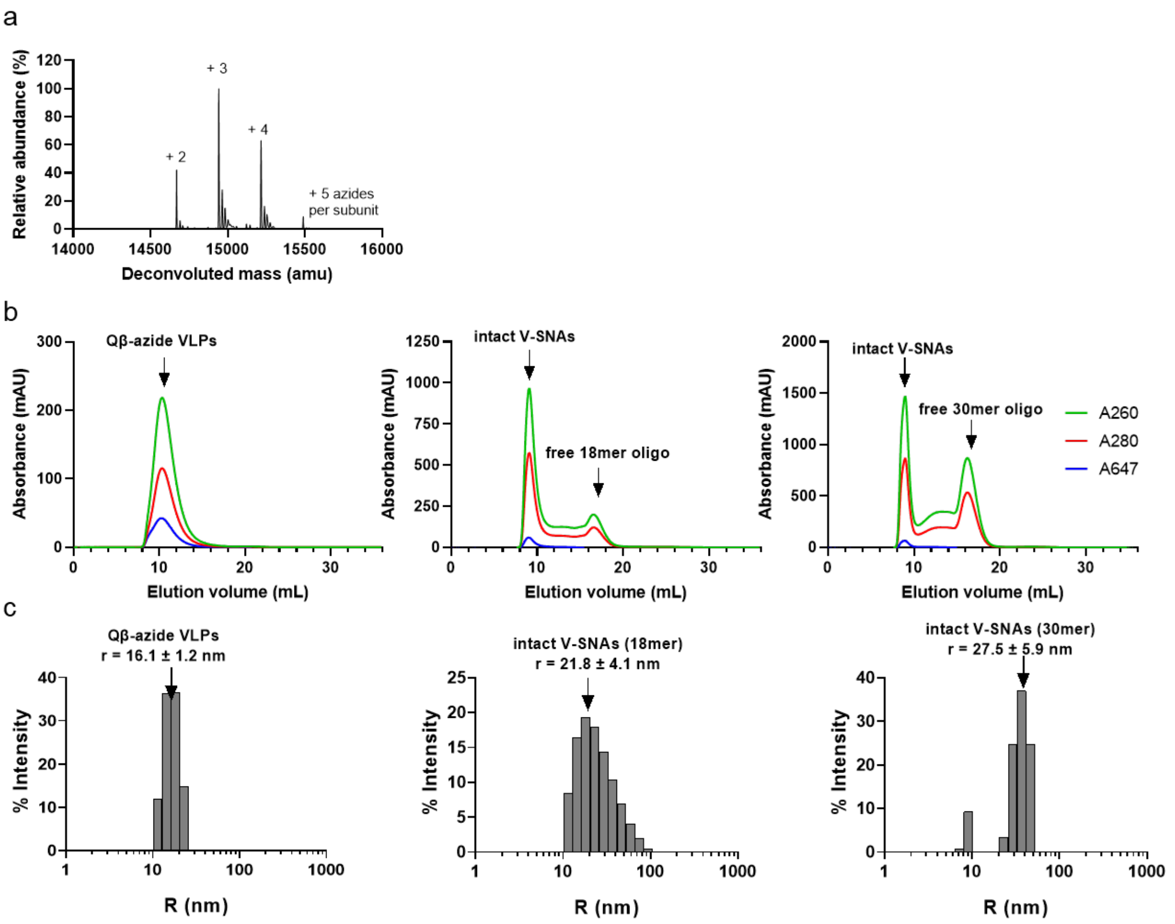

Figure S1. Representative characterization data for azide-modified VLPs and V-SNAs. (a) LC-MS analysis of Qβ VLPs bearing an average of ~560 azides per particle (~3.1 azides per subunit) and (b) Size-exclusion chromatography of Qβ-(azide)$_{550}$ VLPs and V-SNAs; intact VLPs and free oligonucleotides were well-separated on a Superose-6 column (flow rate = 0.4 mL/min, 0.1 M potassium phosphate buffer, pH 7.4). (c) DLS analysis of VLP-azide or V-SNAs.

Ligand protection of oligonucleotides during CuAAC.

Figure S2. Agarose gel electrophoresis of oligonucleotide-coumarin conjugation reactions with CuAAC ligands. Conditions: 0.1 mM Cu, 0.2 mM ligand, 0.2 mM oligonucleotide-alkyne, 0.1 mM coumarin azide,10 mM ascorbate. Loading approx. 1000 ng of nucleic acid per lane, without purification, after 1 h reaction.

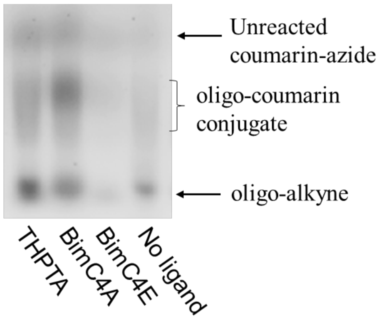

Figure S3. (a) Agarose gel electrophoresis of oligonucleotide-ASGPRL conjugation reactions with CuAAC ligands. Conditions: 0.1 mM Cu, 0.2 mM ligand, 0.2 mM oligonucleotide-alkyne, 0.5 mM ASGPRL-azide, 20 mM ascorbate. Loading: approx. 1500 ng of nucleic acid per lane, without purification, after 1 h reaction (b) Densitometry analysis of gel from panel (a); lanes numbered from left to right.† percent intensity of ASGPR-conjugate relative to total oligo per lane; ‡ percent intensity of oligo per lane relative to lane 8 (untreated) ; n = 2 replicates.

a

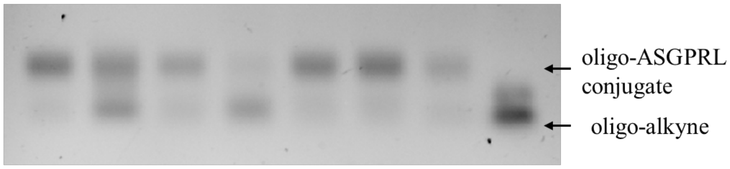

b

Lane	Ligand	Equivs ligand	%-oligo-clicked†	%-oligo-rel.-untreated‡
1	BimC4A	0.5	83.3 ± 3.0	47.4 ± 36.1
2	BimC4A	2.0	64.0 ± 6.8	83.2 ± 32.7
3	BimC4E	0.5	64.3 ± 2.9	33.5 ± 22.7
4	BimC4E	2.0	17.0 ± 8.2	34.1 ± 20.2
5	THPTA	0.5	78.6 ± 2.8	60.5 ± 12.2
6	THPTA	2.0	77.8 ± 0.8	85.0 ± 12.1
7	None	0.0	67.2 ± 1.8	32.0 ± 0
8	None	0.0	NA	100.0

Figure S4. Aminoguanidine and ligand-mediated protection during CuAAC. Conditions: 0.1 mM Cu, 0.2 mM ligand, 0.2 mM oligonucleotide-alkyne, 0.5 mM ASGPRL-azide, 20 mM ascorbate. Loading: approx. 1500 ng of nucleic acid per lane, without purification, after 1 h reaction time.

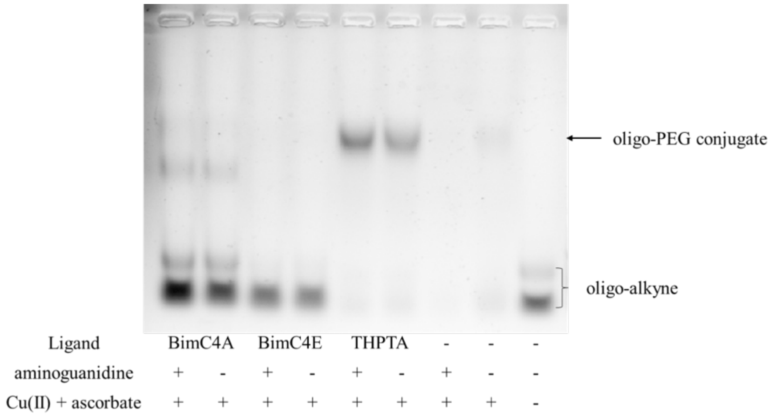

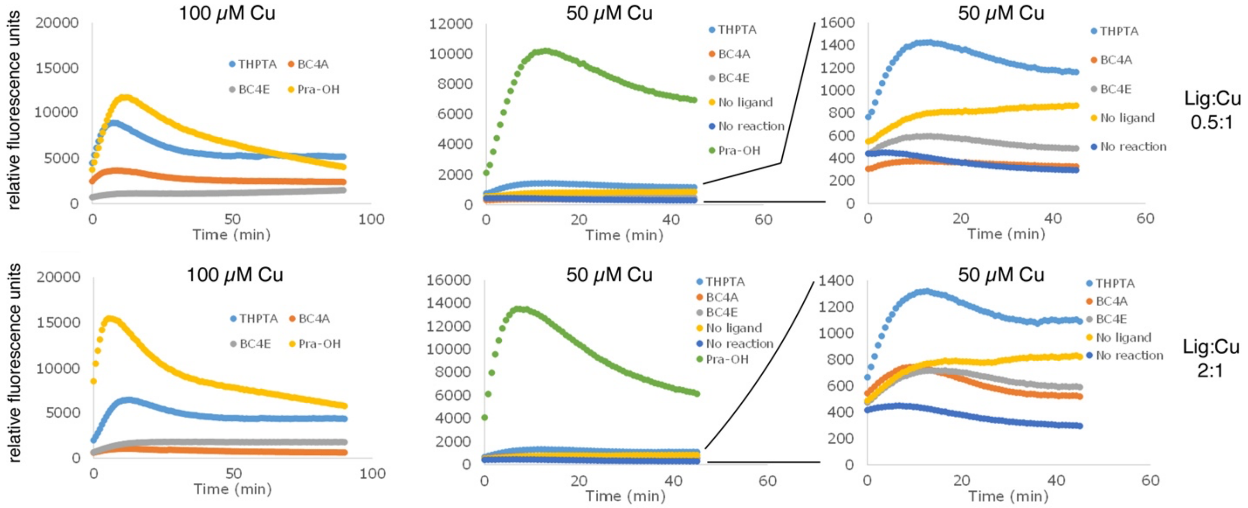

Figure S5. CuAAC between oligonucleotide-alkyne and coumarin azide, assessed via fluorescence of the coumarin triazole product ((λ_{ex} 404 nm, λ_{em} 477 nm).

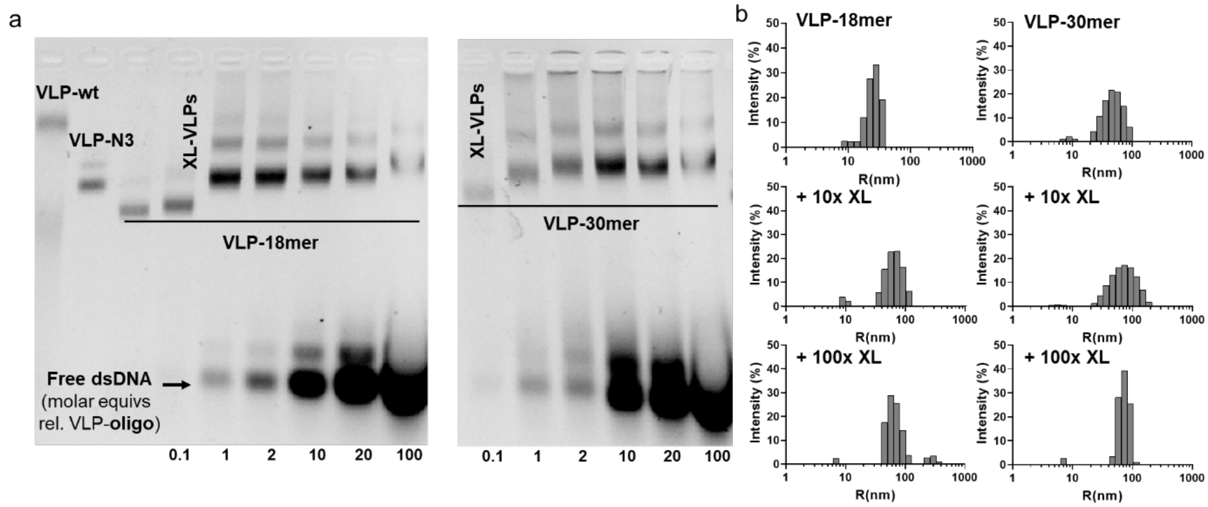

Figure S6. dsDNA-mediated assembly of VLPs. (a) Agarose-gel electrophoresis of VLP-oligonucleotide conjugates. A dsDNA spacer, with 15mer overhang complimentary to VLP-displayed sequences, was added to induce VLP aggregation. (b) Dynamic light scattering of VLP-oligonucleotide conjugates prior to (top), or following (middle, bottom) addition of indicated amounts of dsDNA cross-linking strands.

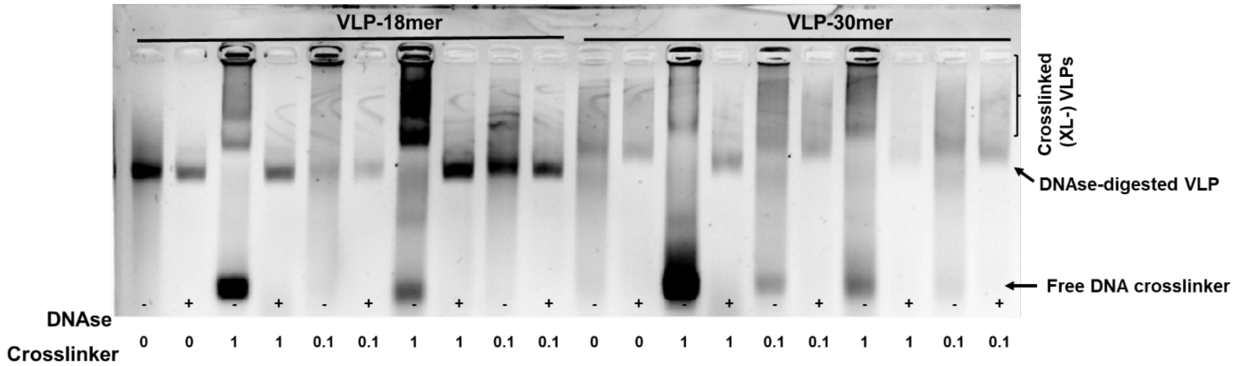

Figure S7. DNase-mediated disassembly of crosslinked VLP. (a) Agarose-gel electrophoresis of VLP-oligonucleotide conjugates or crosslinked VLP-oligonucleotide conjugates prior to, or following, treatment with 0.1 mg/mL DNase I.

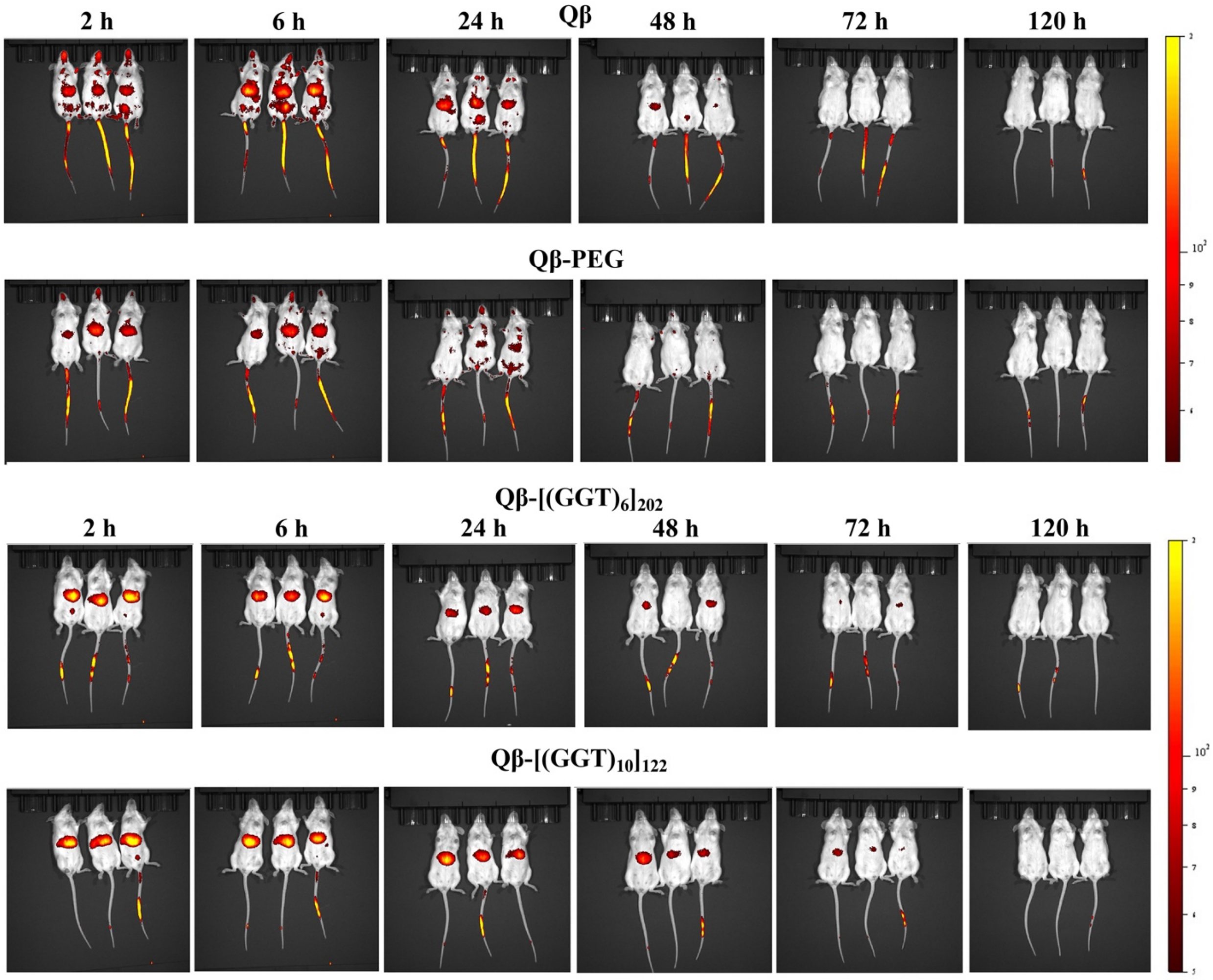

Figure S8. *In vivo* biodistribution images such as those shown in Figure 3.

In vitro microscopy

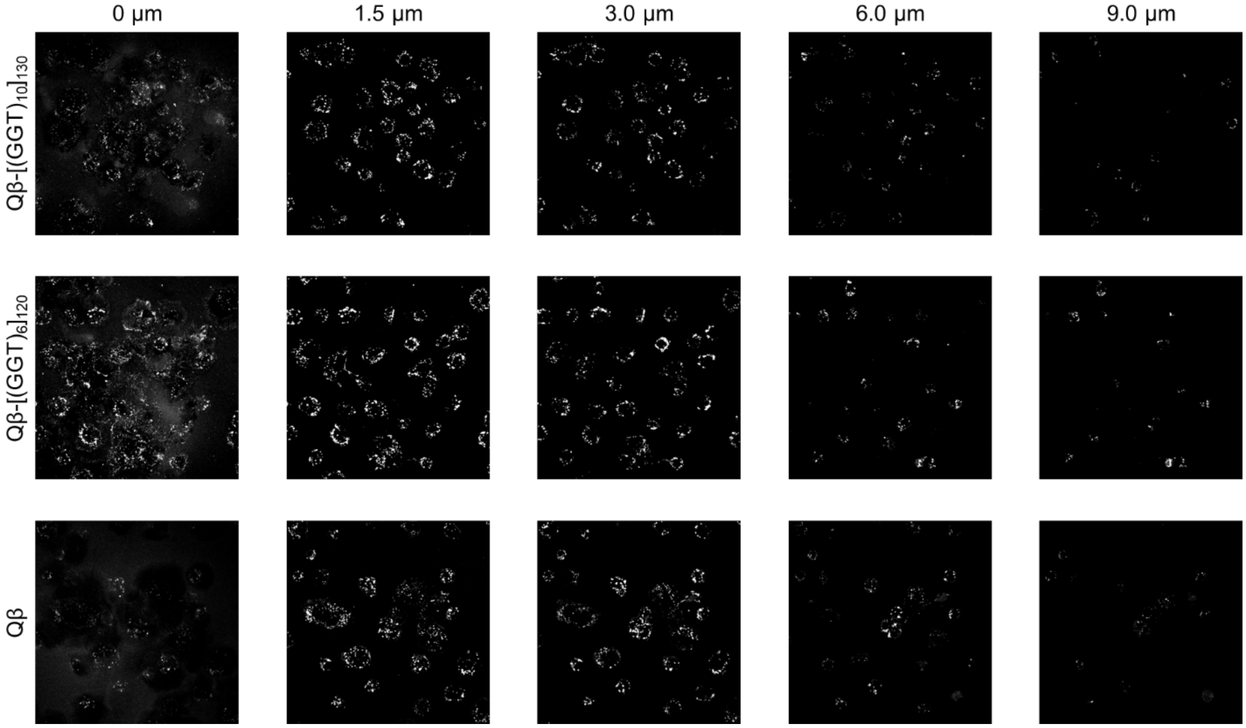

Figure S9. Z-stack image series for C166 treated with 5 nM Qβ-VLPs for 180 minutes (data also shown in Figure 4a). The '0 µm' position was selected based on VLP-binding to the bottom of the plate. The contrast in the 'Qβ' image series was adjusted independently.

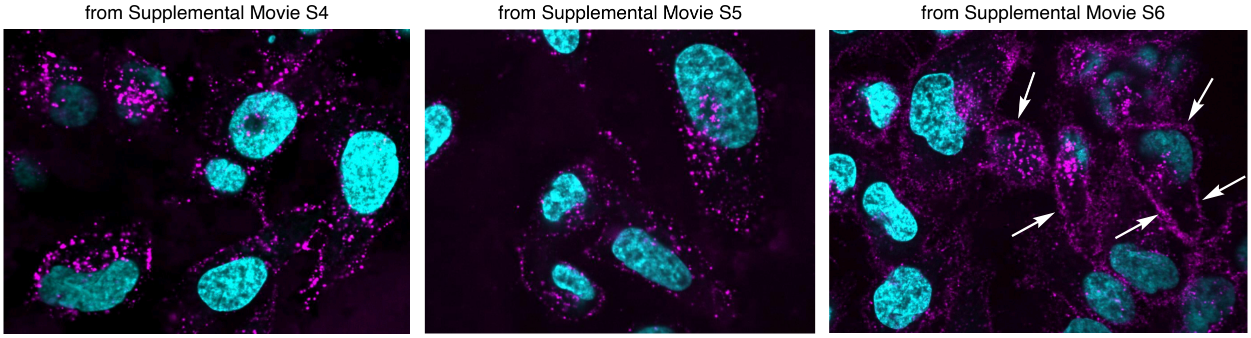

Figure S10. Images taken from z-stack analysis of HeLa cells treated with 5 nM Qβ-VLPs for 180 minutes, as described above for Supplemental Movies S4-S6. In the right-hand image (unmodified Qβ-VLPs), arrows point to examples of staining of the outer cell surface by the particles, a pattern that is not observed with DNA-decorated VLPs; the contrast in the right-hand image was adjusted independently.

Supplemental Movies S1-S3: Z-stack image series of C166 cells treated with 5 nM Qβ-VLPs for 180 minutes, followed by washing with PBS and treatment with Lysotracker (100 nM) and Hoescht (10 μg/mL). Image stacks show signal from nuclear (cyan), lysosomal (yellow), and VLP (magenta) staining.

Supplemental Movies S4-S6: Z-stack image series of HeLa cells treated with 5 nM Qβ-VLPs for 180 minutes, followed by washing with PBS and treatment with Hoescht (10 μg/mL). Image stacks show signal from nuclear (cyan) and VLP (magenta) staining.
